# Effects of resistance exercise on endothelial progenitor cell mobilization in women

**DOI:** 10.1038/s41598-017-18156-6

**Published:** 2017-12-19

**Authors:** Fernando Ribeiro, Ilda P. Ribeiro, Ana C. Gonçalves, Alberto J. Alves, Elsa Melo, Raquel Fernandes, Rui Costa, Ana B. Sarmento-Ribeiro, José A. Duarte, Isabel M. Carreira, Sarah Witkowski, José Oliveira

**Affiliations:** 1 School of Health Sciences and Institute of Biomedicine - iBiMED, University of Aveiro, Aveiro, Portugal; 2 Center of Investigation on Environment Genetics and Oncobiology (CIMAGO), Faculty of Medicine, University of Coimbra, and Center for Neuroscience and Cell Biology and Institute for Biomedical Imaging and Life Sciences (CNC.IBILI), Coimbra, Portugal; 3 Laboratory of Oncobiology and Hematology, University Clinic of Hematology and Applied Molecular Biology, Faculty of Medicine, and Clinical Hematology Department, Centro Hospitalar Universitário de Coimbra (CHUC), Coimbra, Portugal; 4 Research Centre in Sports Sciences, Health and Human Development, CIDESD, University Institute of Maia, ISMAI, Maia, Portugal; 5 School of Health Sciences, University of Aveiro, Aveiro, Portugal; 6 School of Health Sciences and CINTESIS.UA, University of Aveiro, Aveiro, Portugal; 7 Hematology Department, Centro Hospitalar Universitário de Coimbra, Coimbra, Portugal; 8 Research Center in Physical Activity, Health and Leisure, CIAFEL, Faculty of Sport, University of Porto, Porto, Portugal; 9 Department of Kinesiology, University of Massachusetts Amherst, Amherst, Massachusetts United States of America

## Abstract

This study aimed to determine the effect of a single bout of resistance exercise at different intensities on the mobilization of circulating EPCs over 24 hours in women. In addition, the angiogenic factors stromal cell-derived factor 1 (SDF-1α), vascular endothelial growth factor (VEGF), hypoxia-inducible factor 1-alpha (HIF-1α) and erythropoietin (EPO) were measured as potential mechanisms for exercise-induced EPCs mobilization. Thirty-eight women performed a resistance exercise session at an intensity of 60% (n = 13), 70% (n = 12) or 80% (n = 13) of one repetition maximum. Each session was comprised of three sets of 12 repetitions of four exercises: bench press, dumbbell curl, dumbbell squat, and standing dumbbell upright row. Blood was sampled at baseline and immediately, 6 hours, and 24 hours post-exercise. Circulating EPC and levels of VEGF, HIF-1α and EPO were significantly higher after exercise (P < 0.05). The change in EPCs from baseline was greatest in the 80% group (P < 0.05), reaching the highest at 6 hours post-exercise. The change in EPCs from baseline to 6 hours post-exercise was correlated with the change in VEGF (r = 0.492, P = 0.002) and HIF-1α (r = 0.388, P = 0.016). In general, a dose-response relationship was observed, with the highest exercise intensities promoting the highest increases in EPCs and angiogenic factors.

## Introduction

Endothelial progenitor cells (EPCs), are circulating precursors of endothelial cells derived from the bone marrow, that have the ability to promote endothelial repair, neovascularization and the restoration of endothelial function^1–3^. Endothelial regeneration promoted by EPCs is achieved either by their incorporation into the blood vessels and differentiation into mature endothelial cells or by stimulating mature endothelial cells to proliferate via a paracrine mechanism^2,4^. The number and function of EPCs are positively associated with vascular function, inversely correlated with cardiovascular risk factors^5,6^ and may, ultimately, predict cardiovascular events and death^7^. The number of EPCs in circulation is very low, therefore their mobilization from the bone marrow is a paramount step to promote endothelial repair following chemical or mechanical injury.

Physical exercise is one of the most important physiological stimuli to mobilize EPCs both in healthy individuals and patients with coronary artery disease. Several studies show increased number of circulating EPCs after an acute bout of aerobic^8^, particularly strenuous exercise^9–11^. Likewise, aerobic exercise training also improves the number of resting circulating EPCs in healthy subjects and patients with cardiovascular disease^1,8^. Further, reductions in physical activity reduce the number of select angiogenic cell populations^12^, demonstrating that characteristics of physical activity are important for the liberation of these cells into circulation.

Compared to aerobic exercise, there are few studies evaluating the impact of resistance exercise on circulating EPCs^13,14^. Further, the majority of the available evidence comes from studies that have enrolled only men^9,13–16^. Ross *et al*. showed that short bouts of muscular endurance resistance exercise increase the number of circulating EPCs of trained men^14^; similarly, Kurger *et al*.^13^ reported greater circulating EPCs, in men aged 21–29 years old, after resistance exercise performed at 75% of 1 repetition maximum (1-RM). Evaluation of resistance exercise-induced mobilization of EPCs in women is warranted because previous studies have shown that men and women exhibit different immune^17^, circulating angiogenic cell^18^ and circulating endothelial cell-derived microparticle^19^ response to exercise.

Resistance exercise characteristics, namely duration and intensity of exercise appear to impact the degree of mobilization of EPCs from bone marrow. For example, Laufs *et al*.^15^ compared the effects of three protocols of running exercise, 30 minutes at 82% maximal oxygen consumption (VO_2max_), 30 minutes at 68% VO2max and 10 minutes at 68% VO_2max_, on the number and function of circulating EPCs in 25 healthy, male volunteers. Only the two 30-minute running bouts increased circulating levels of EPC, irrespective of intensity. It is also not known whether the intensity of the resistance exercise directly affects the number of circulating EPCs and whether a dose-response relationship exists. Thus, the purpose of this study was to determine the influence of a single bout of resistance exercise at different intensities on the mobilization of circulating EPCs over 24 hours in women. Further, angiogenic factors associated with exercise-induced EPCs mobilization, namely stromal cell-derived factor 1 (SDF-1α), vascular endothelial growth factor (VEGF), hypoxia-inducible factor 1-alpha (HIF-1α) and erythropoietin (EPO), were evaluated and correlated with changes in EPCs.

## Results

### Participant characteristics

The characteristics of the participants are summarized in Table 1. Groups were statistically similar at baseline for all variables, including age, weight, body mass index and circulating levels of EPCs. There were no baseline differences between groups in physical characteristics, circulating EPCs and angiogenic factors (Table 1) indicating relative balance between the assigned groups.

**Table 1:** Baseline characteristics of the participants.

N	**60% 1-RM**	**70% 1-RM**	**80% 1-RM**	**P value**
	13	12	13	—
Age (years)	20.7 ± 1.7	21.0 ± 0.9	20.9 ± 1.4	0.842
Weight (kg)	60.2 ± 5.0	58.2 ± 12.2	61.4 ± 8.1	0.666
Height (m)	1.64 ± 0.06	1.64 ± 0.08	1.65 ± 0.06	0.923
BMI (kg/m^2^)	22.4 ± 2.4	21.4 ± 2.5	22.6 ± 3.5	0.536
EPCs (%)	8.69E^−03^ ± 0.97E^−03^	7.65E^−03^ ± 0.42E^−03^	7.87E^−03^ ± 0.37E^−03^	0.505
EPO (mU/mL)	10.1 ± 3.7	12.4 ± 7.6	11.7 ± 4.9	0.588
HIF-1α (pg/mL)	322.9 ± 203.7	320.4 ± 240.9	321.6 ± 179.3	1.000
SDF-1 (pg/mL)	1312.6 ± 486.5	1377.2 ± 343.1	1327.3 ± 471.4	0.930
VEGF (pg/mL)	52.0 ± 6.5	51.7 ± 6.3	52.4 ± 8.9	0.971

BMI, body mass index; EPCs, endothelial progenitor cells; EPO, erythropoietin; SDF-1α, stromal cell-derived factor 1; VEGF, vascular endothelial growth factor; HIF-1α, hypoxia-inducible factor 1-alpha and EPO, erythropoietin; P values are for between group comparisons with one-way ANOVA.

### Effects on EPCs

**Figure 1:**
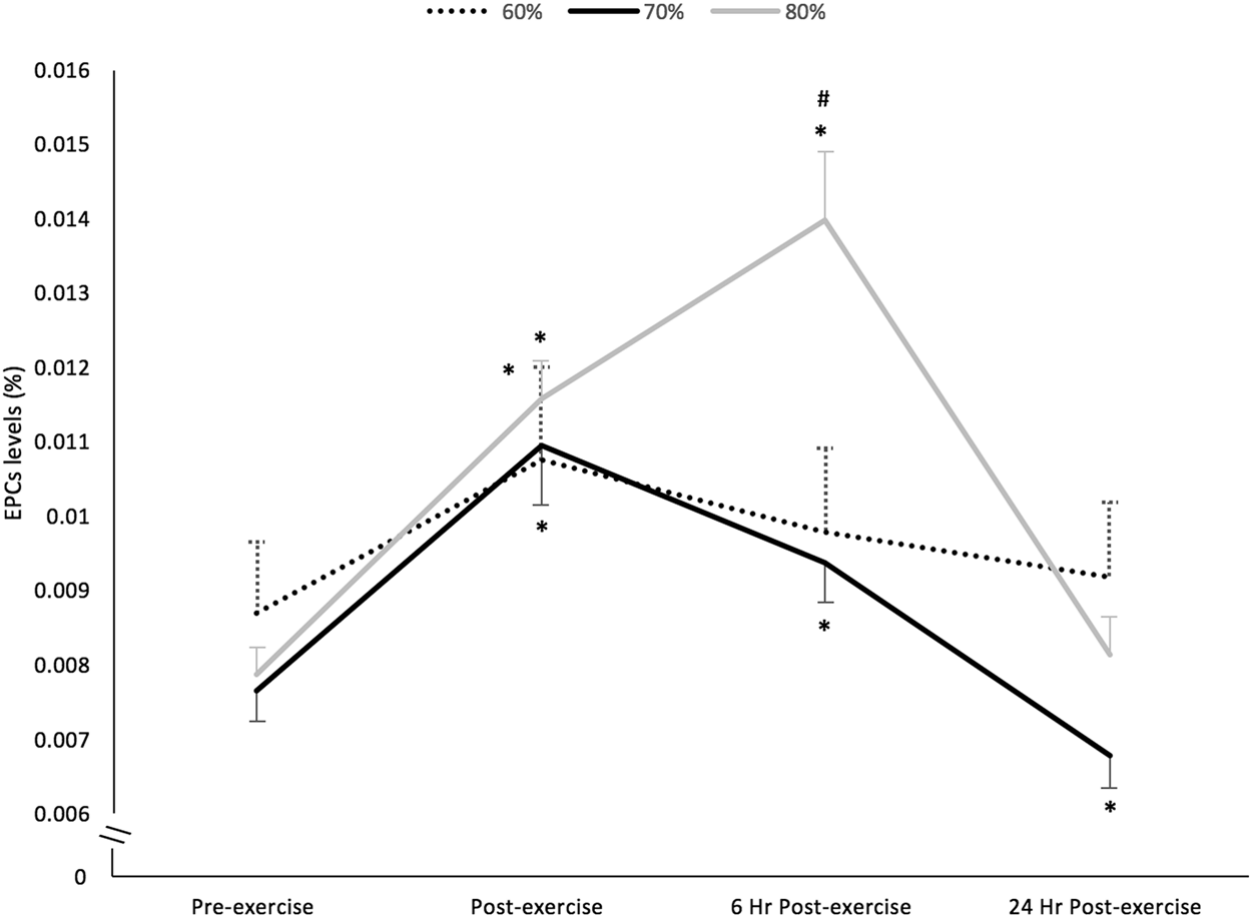
Influence of an acute bout of resistance exercise at different intensities on EPCs levels (mean ± SEM). *P < 0.05 compared with pre-exercise. ^#^P < 0.01 compared with exercise at 60% and 70% 1-RM.

The three exercise intensities induced different effects on EPCs over time (group X time interaction, F_6,66_ = 11.776; P < 0.001; η^2^_p_ = 0.517). The circulating levels of EPCs changed significantly over time (F_3,36_ = 22.634; P < 0.001; η^2^_p_ = 0.654) after 80% 1-RM exercise; EPC levels were significantly greater post-exercise (baseline 7.87E^−03^ ± 0.37E^−03^ to 11.58E^−03^ ± 0.52E^−03%^, P < 0.001), reached the highest at 6 hours post-exercise (13.97E^−03^ ± 0.93E^−03%^, P < 0.001) and returned to baseline values 24 hours post-exercise (8.14E^−03^ ± 0.51E^−03%^, P > 0.05) (Fig. 1). Resistance exercise at 60% 1-RM (F_3,36_ = 10.040; P < 0.001; η^2^_p_ = 0.456) and 70% 1-RM (F_3,33 = _33.273; P < 0.001; η^2^_p_ = 0.752) also promoted a significant change in the circulating levels of EPCs over time. In the 60% 1-RM exercise group, circulating EPC levels were significantly greater only post-exercise (baseline 8.69E^−03^ ± 0.97E^−03^ to 10.75E^−03^ ± 1.25E^−03^%, P = 0.038). In the 70% 1-RM group circulating EPCs were significantly greater post-exercise (baseline 7.65E^−03^ ± 0.42E^−03^ to 10.95E^−03^ ± 0.81E^−03%^, P < 0.001) where they reached the peak value, and at 6 hours post-exercise (9.38E^−03^ ± 0.54E^−03%^, P = 0.001) and decreased to values below baseline after 24 hours post-exercise (6.79E^−03^ ± 0.44E^−03^%, P = 0.029) (Fig. 1).

All the three exercise intensities promoted a significant increase in circulating EPCs immediately post-exercise, nonetheless the change in EPCs levels was higher in the session at 80% 1-RM compared with 60% 1-RM (49.3 ± 7.2 vs. 24.3 ± 5.6%, P = 0.019). Resistance exercise at 80% 1-RM also induced a change in circulating EPCs from pre-exercise to 6 hours post-exercise that was higher than both 60% 1-RM (81.1 ± 13.6 vs. 12.7 ± 4.3%, P < 0.001) and 70% 1-RM (81.1 ± 13.6 vs. 23.0 ± 3.9%, P < 0.001).

### Effects on angiogenic factors

**Figure 2:**
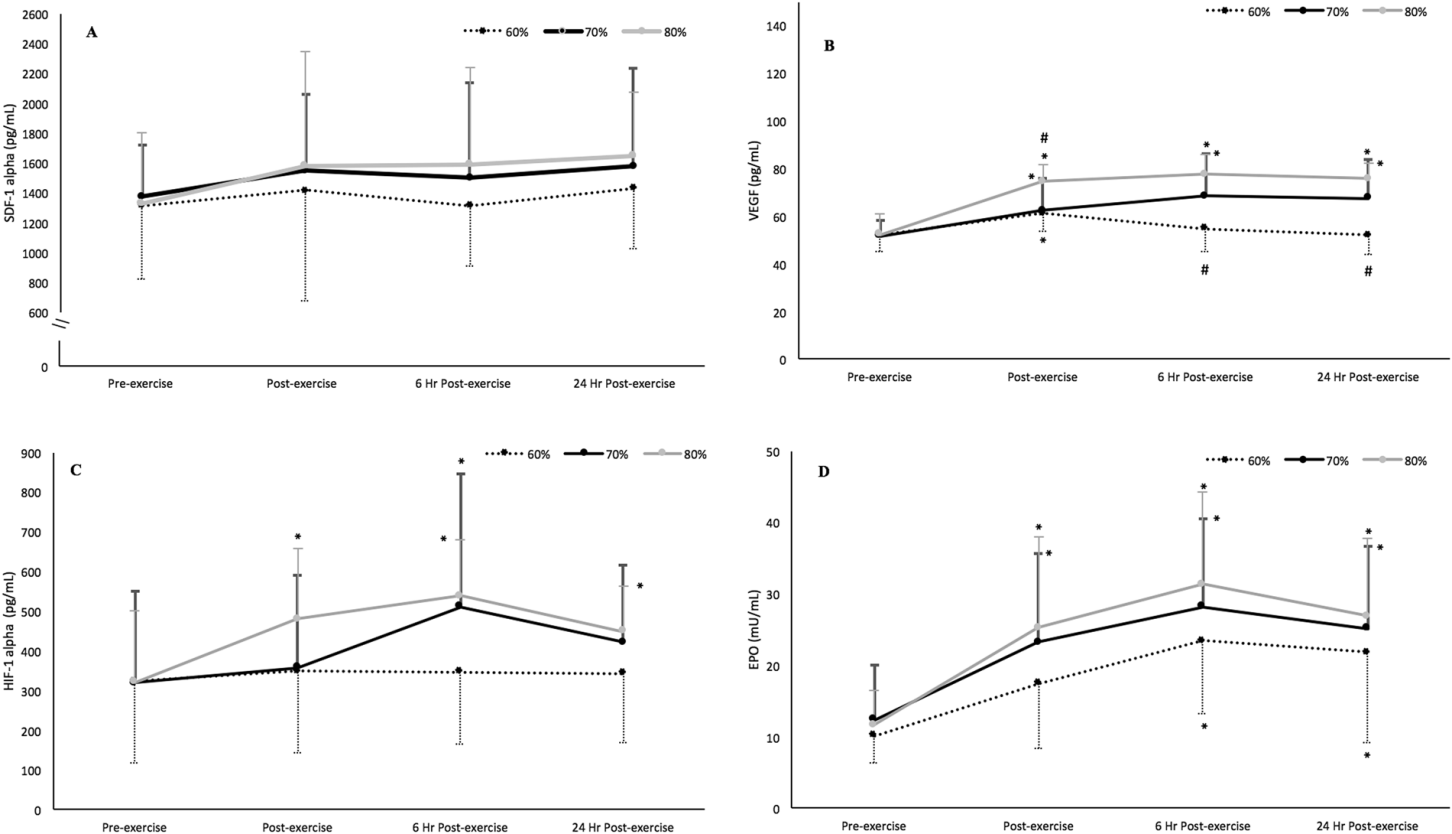
Influence of an acute bout of resistance exercise at different intensities on 24-hr circulating SDF-1α (**A**), VEGF (**B**), HIF-1α (**C**), and EPO (**D**) (mean ± SEM). *P < 0.05 compared with pre-exercise; ^#^P < 0.01 compared with the other two exercise intensities.

No differences were observed in plasma SDF-1α in any group; SDF-1α did not change significantly over time (F_3,33_ = 0.795; P = 0.506; η^2^_p_ = 0.067) and there was no observed interaction for group X time (F_6,66_ = 0.643; P = 0.643; η^2^_p_ = 0.055) (Fig. 2A).

Conversely, VEGF levels changed significantly over time (F_3,33_ = 27.901; P < 0.001; η^2^_p_ = 0.717), with a significant interaction for group X time (F_6,66_ = 8.124; P < 0.001; η^2^_p_ = 0.428). The 70% 1-RM and 80% 1-RM groups showed a significantly higher VEGF immediately, at 6 and 24 hours post-exercise compared with the values at baseline (Fig. 2B). In the 60% 1-RM group, differences were observed only immediately after exercise in comparison with baseline (52.0 ± 6.5 vs. 61.2 ± 7.4 pg/mL, P = 0.039). Baseline to post-exercise changes were significantly higher in the 80% 1-RM group in comparison to the 60% 1-RM group (44.2 ± 15.3 vs. 19.1 ± 19.6%, P = 0.003) and 70% 1-RM group (44.2 ± 15.3 vs. 20.3 ± 18.8%, P = 0 0.006); baseline to 6 hours post-exercise changes were lower in the 60% 1-RM session per comparison to both 70% 1-RM (5.3 ± 4.0 vs. 33.2 ± 9.2%, P = 0.017) and 80% 1-RM (5.3 ± 4.0 vs. 50.6 ± 6.0%, P < 0.001) sessions; baseline to 24 hours post-exercise changes were also lower in the 60% 1-RM session per comparison to both 70% 1-RM (1.3 ± 5.3 vs. 30.1 ± 6.7%, P = 0.008) and 80% 1-RM (1.3 ± 5.3 vs. 47.8 ± 6.6%, P < 0.001) sessions.

Plasma HIF-1α, changed significantly over time (F_3,33_ = 3.024; P = 0.042; η^2^_p_ = 0.20), but no significant interaction for group X time (F_6,66_ = 1.292; P = 0.272; η^2^_p_ = 0.097) was observed (Fig. 2C). When comparing percentage changes from baseline to post-exercise, the 80% 1-RM group was significantly greater compared to both 60% 1-RM (68.0 ± 57.0% vs. 12.8 ± 24.2%, P = 0.015) and 70% 1-RM groups (68.0 ± 57.0% vs. 20.7 ± 52.8%, P = 0.048). The percent change from baseline to 6 hours post-exercise was lower in the 60% 1-RM group compared to both 70% 1-RM (15.0 ± 25.6% vs. 70.3 ± 53.5%, P = 0.036) and 80% 1-RM groups (15.0 ± 25.6% vs. 96.8 ± 68.3%, P = 0.001).

Plasma EPO changed significantly over time (F_3,33_ = 4.816; P = 0.007; η^2^_p_ = 0.305), with a significant group X time interaction (F_6,66_ = 9.314; P < 0.001; η^2^_p_ = 0.459). Plasma EPO increased significantly in all groups at all time points in comparison to baseline, with the exception of the 60% 1-RM group immediately after exercise (mean difference, 95% CI: 7.38, −0.36–15.11 mU/mL; P = 0.065) (Fig. 2D). Percentage change in EPO from baseline to post-exercise (P = 0.515), baseline to 6- (P = 0.753) and 24-hours post-exercise (P = 0.900) was similar among groups.

### Associations between changes in EPCs and angiogenic factors

**Figure 3:**
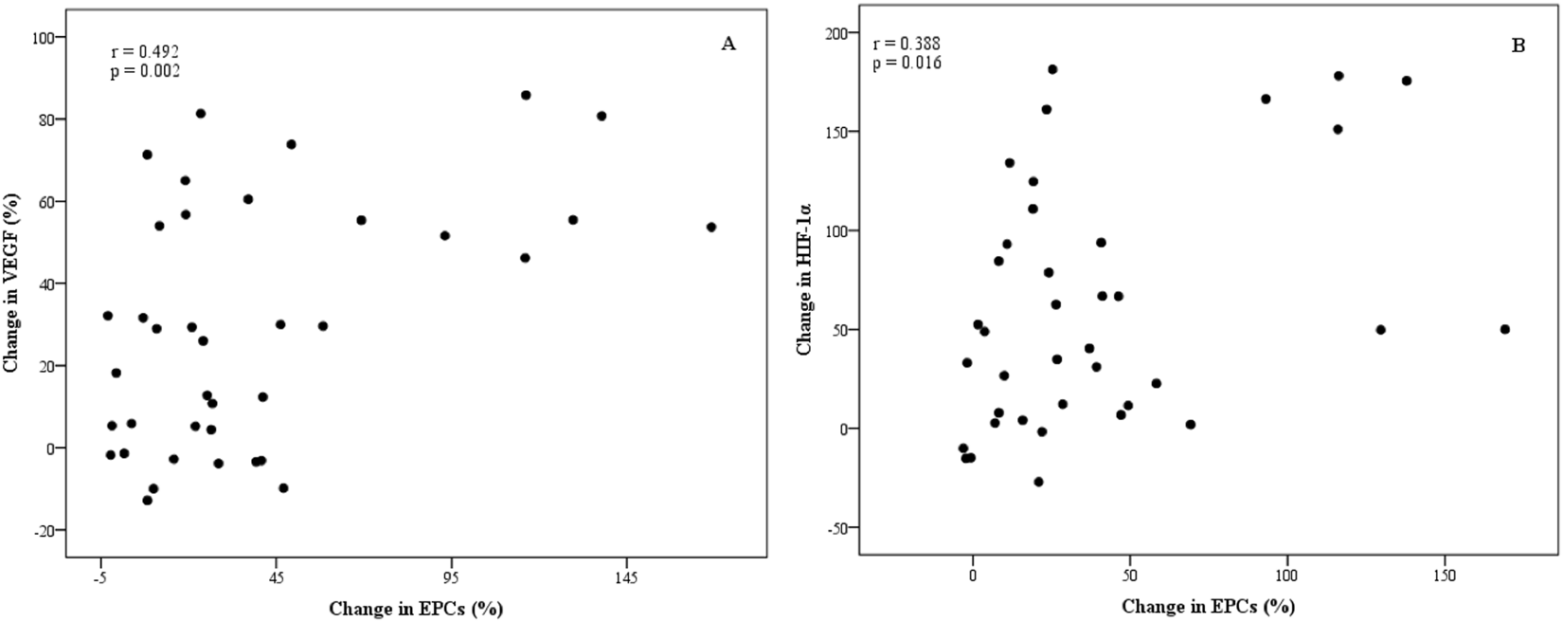
Correlations of exercise-induced change in EPCs from baseline to 6 hours post-exercise with change in VEGF (**A**) and HIF-1α (**B**) from baseline to 6 hours post-exercise.

The change in EPCs from baseline to 6 hours post-exercise was significantly correlated with the change in VEGF (r = 0.492, P = 0.002; Fig. 3A) and HIF-1α (r = 0.388, P = 0.016; Fig. 3B). No further correlations were found.

## Discussion

This study describes the effect of a single resistance exercise bout performed at different intensities on circulating levels of EPCs over 24 hours in young women. In addition, the effects of exercise on circulating angiogenic factors EPO, VEGF, HIF-1α and SDF-1α were also measured as potential mechanisms for exercise-induced EPCs mobilization in this population. The data reveal that (i) an acute bout of resistance exercise significantly increases EPCs levels, (ii) the highest exercise intensity tested (80% of 1-RM) induced the greatest increase in circulating EPCs, (iii) the mobilization of progenitor cells seems to be dependent on exercise intensity, (iv) resistance exercise increases the levels of the VEGF, HIF-1α and EPO, (v) the exercise-induced increase of EPCs is positively associated with changes in VEGF and HIF-1α levels. To our best knowledge, this is the first study to demonstrate a dose-response relationship between resistance exercise intensity and the circulating levels of EPCs in women.

Our results are similar to those observed by Ross *et al*.^14^ and Kruger *et al*.^13^ who also defined EPC as CD45^dim^/VEGFR2^+^/CD34^+^ and reported a significant increase in EPCs at 2^14^ or 3 hours^13^ after resistance exercise. However, we observed a significant increase immediately after exercise, which returned to baseline 24 hours post exercise. In contrast, in the study of Kruger *et al*.^13^ the values peaked at 24 hours and returned to baseline at 48 hours post-exercise. Also, the magnitude of EPCs increase in our study (81.1 ± 13.6% at 6 hours postexercise at 80% 1-RM) was similar to the increase reported by Ross *et al*.^14^ (approximately 60–65%) but lower than that obtained by Kruger *et al*.^13^ (approximately 300%). These differences could be related with the exercise stimuli as the current study employed a resistance exercise protocol while Ross *et al*.^14^ tested a muscular endurance resistance exercise protocol, with three circuits of 15 repetitions of six exercises using light to moderate weights with little recovery between repetitions or exercises. Further, Kruger *et al*.^13^ employed a resistance exercise protocol (at 75% of 1 RM) with a duration 3 times higher than ours with twice the exercises. Taken together, these results indicate that the kinetics of EPC mobilization vary with the intensity characteristics of the resistance exercise.

Mobilization of EPC has been investigated to a greater extent with endurance exercise^9,10,20^. Fewer have evaluated whether resistance exercise can mobilize EPCs^13,14^. Further, we are one of the first to evaluate EPC mobilization in response to resistance exercise in women. Despite the evidence supporting the benefits of exercise training to prevent and treat disease in both men and women, women are often underrepresented in exercise and sports science research^21^, creating significant knowledge gaps on sex differences in responses to exercise. Additionally, it is documented that the responses to resistance exercise are different between men and women^22,23^, but to date no studies existed determining the EPC response to resistance exercise in women. Shill *et al*.^18^ recently reported differences in various circulating angiogenic cell types between men and women in response to acute maximal endurance exercise, however they did not evaluate EPC as defined herein. Further, they reported variation in select cell populations by menstrual cycle phase and contraceptive use. Therefore, evaluation of sex differences and variation among sexes in the response to acute exercise and exercise training is necessary.

It has been suggested that brief reversible ischemic stimuli and hypoxia play a vital role in triggering VEGF-mediated mobilization of EPCs^24,25^. In fact, the transient cellular hypoxia and increased shear stress that occurs during exercise induces the upregulation of VEGF, SDF-1α and EPO, among others angiogenic factors, mediated by HIF-1α and nitric oxide, which in turn stimulate EPCs mobilization^8^. HIF-1α plays a particularly important role as it activates the transcription of VEGF, SDF-1α, and EPO^4,8^; during episodes of reduced tissue oxygen tension the levels of HIF-1α tend to increase, thus activating SDF-1α^26^. Previous studies have shown that after aerobic exercise the expression and stabilization of HIF-1α in skeletal muscle are enhanced^27,28^. In addition to EPCs mobilization, HIF-1α also promotes EPCs function and inhibits apoptosis^29^. In the present study, the increase in EPCs was accompanied by an increase in VEGF, EPO and HIF-1α, but not by an increase in SDF-1α. Our results contrast with a recent study that submitted 5 healthy volunteers to a 30-minute run and observed a significant increase in plasma levels of SDF-1α after exercise^30^. Contrary to others^14^, we observed a positive correlation between EPC mobilization and increases in the angiogenic factors VEGF and HIF-1α. Previous studies have also highlighted the role of SDF-1α in progenitor cell recruitment and effectiveness of stem cell therapy after a myocardial infarction^31–33^. Nonetheless, the lack of association, in our study, between changes in EPCs and EPO, the weak correlations between EPCs and VEGF and HIF-1α, together with the absence of significant changes in SDF-1α, seem to indicate that the resistant exercise-mediated mobilization of EPCs is a collaborative response and the importance of any single angiogenic factor is relative. Future studies should consider the inclusion of a broader panel of angiogenic markers and related pathways.

Future work should address whether the acute improvement in EPCs observed after the bouts of resistance exercise is accompanied by a better endothelial function. A recent systematic review and dose–response meta-analysis showed that resistance exercise training improves endothelial function and that greater frequency, rather than intensity, is the key factor to maximize improvement in flow-mediated dilation^34^; per opposition to the chronic effects, the acute effects of resistance exercise on endothelial function are not consensual. Some evidence has shown that acute high-intensity resistance eccentric exercise^35^ may decrease endothelial function, whereas dynamic submaximal resistance exercise seems to acutely improve endothelial function in hypertensive rats^36,37^, type 2 diabetes patients^38^ and healthy males^39^.

### Study limitations

The current study is not without limitations. First, the effect of acute resistance exercise was assessed immediately after exercise and then 6 and 24 h post-exercise. Since peak circulating EPC at 60% and 70% 1-RM exercise bouts was immediately following exercise, future studies should assess EPCs liberation between immediately and 6 hours after exercise to clarify the time course of peak of EPCs mobilization induced by different resistance exercise intensities in women. Second, the between-group design is a limitation however, this design was chosen to eliminate potential carryover effects from the different conditions or determination of appropriate washout period inherent in a within-group design. Another limitation of the study is that the participants were not traditionally randomized, but were assigned consecutively to each group. Nevertheless, the potential bias associated with the allocation of the participants is limited, since no baseline differences between groups were observed in any variable. Fourth, we did not strictly control for menstrual cycle phase which may have underestimated the effects of the exercise; some studies reported variation in circulating angiogenic cells^18^, progenitor cells and angiogenic factors during menstrual cycle^40^; nonetheless we found significant changes in EPCs despite variations in menstrual cycle timing. Finally, since all participants included into this study were non-trained healthy women, it is not clear if the same effect would be observed in well-trained subjects or in patients with cardiovascular disease. Hence, it will be important to determine if there are sex differences in the responses to exercise for trained and patient populations. It is well known that endurance exercise mobilizes EPCs in patients with cardiovascular disease, understanding whether resistance exercise mobilizes EPCs and the nature of the response can increase the evidence for the mechanism by which different exercise modes influence vascular homeostasis in men and women.

In conclusion, the results of this study show that a short bout of resistance exercise promotes an acute increase in circulating EPCs in women; this mobilization of EPCs is accompanied by increases in VEGF, EPO and HIF-1α. In general, a dose-response relationship was observed both in EPCs and in the angiogenic factors, with the highest exercise intensities promoting the highest increases in the circulating levels of EPCs, VEGF and HIF-1α. Our findings highlight the efficacy of resistance exercise in women to enhance neoangiogenesis and repair of endothelial cell layer.

## Methods

### Study population and design

The effect of a single bout of resistance exercise at different intensities on circulating EPCs was studied in 38 healthy, premenopausal female participants. Women were between the ages 18 and 30 years, experienced regular menstrual cycles and were not taking contraceptives. Participants did not participate in regular exercise (<2 times per week), were not using dietary supplements and had no history of resistance exercise training in the last year. Recruitment occurred through verbal advertisement, email, and posting the invitation on online social media. The following exclusion criteria were applied: any significant medical history (e.g. cardiovascular disease, metabolic disease, cancer), smoking, pharmacologic treatment for any condition, and musculoskeletal injuries precluding exercise.

Exercise sessions and data collection occurred outside the menstrual period phase of the menstrual cycle (i.e. days 1–5). All participants completed two study visits, once for assessment of muscle strength to determine the 1- repetition maximum for each exercising muscle group, and once (5 to 7 days after the first visit) to perform the resistance exercise session at an intensity of 60% (n = 13), 70% (n = 12) or 80% (n = 13) of 1-RM. Participants meeting the inclusion criteria and willing to participate were consecutively allocated to one of three groups; i.e. the first 13 participants accepting to participate were allocated to the 80% group; the participants 14 to 26 were allocated to the group of 60% and the last group of 13 to the group of 70%. One participant in the 70% group dropped out after the assessment of muscle strength leaving this group with an n = 12. This type of allocation was used to minimize carry over effects between exercise intensities. To determine the acute response of resistance exercise on circulating EPCs, venous blood was sampled immediately before, immediately after (within 5 min cessation of exercise), 6 h, and 24 h after exercise. Plasma and serum samples were collected and stored at −80 °C to further determine the circulating levels of SDF-1α, VEGF, HIF-1α and EPO.

The ethics committee of the Faculty of Sport, University of Porto, approved the studies (CEFADE 31.2014). All participants provided written informed consent and all procedures were conducted according to the Declaration of Helsinki.

### Muscular resistance exercise

Five to seven days before the exercise session, all participants performed a muscle strength assessment to determine 1-RM in each exercise that was going to be performed in the exercise session. An experienced researcher assessed the 1-RM for the following exercises: barbell bench press, standing dumbbell curl, dumbbell squat, and standing dumbbell upright row. Participants were familiarized with the test and training exercises, and their 1-RM was determined as previously described^41^. The 1-RM was defined as the greatest amount of weight one can lift in a single movement through the full range of motion, in a controlled manner.

The resistance exercise sessions were performed at an intensity of 60%, 70% or 80% of 1-RM and comprised three sets of 12 repetitions of the four exercises mentioned above. The participants rested 1 minute between sets; each exercise session lasted approximately 30 minutes. All the exercise sessions took place in the morning, between 8:30am and 11am.

### EPCs quantification by flow cytometry

Blood samples (3 mL) for EPC analysis were collected into EDTA tubes and treated, according to manufacturer’s instructions, with TransFix (Cytomark, Caltag Medsystems Ltd, Buckingham, UK) at a 1:5 ratio immediately after collection. These blood samples were stored in the dark at room temperature until flow cytometry analysis (at day three or day four after collection). It was demonstrated that it is possible to analyse TransFix stabilized blood cells up to seven days after blood collection^42^.

**Figure 4:**
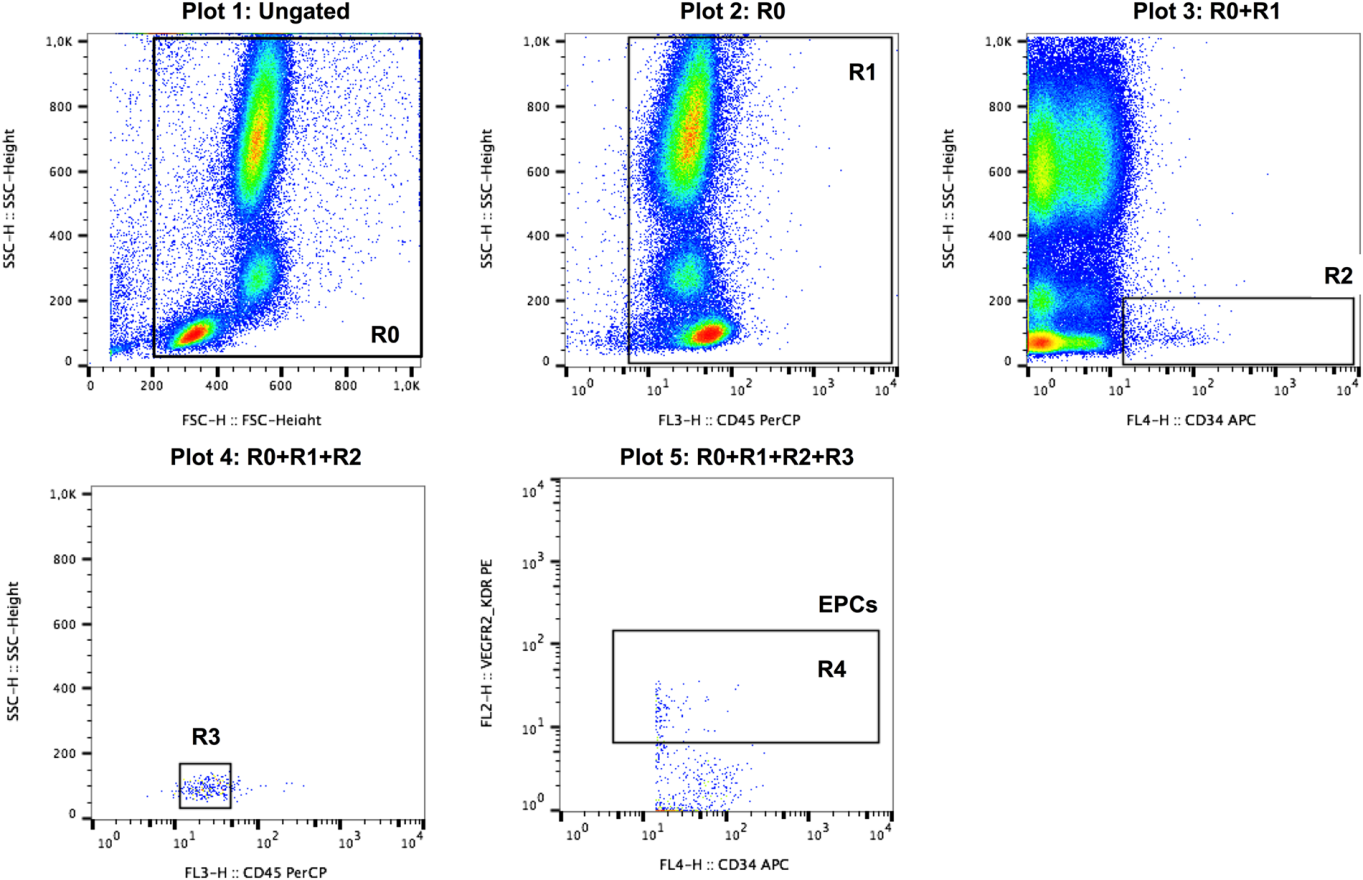
Flow cytometry gating strategy to quantify endothelial progenitor cells (EPCs). Initially, debris, red blood cells and platelets were removed from the analysis based on their forward scatter (FCS) vs. side scatter (SSC) properties (R0 plot 1) and then a gate was set on a CD45 vs. dot plot to contain all CD45^+^ events (R1 plot 2). Next, gate R1 events were displayed on a CD34 vs. SSC dot plot (plot 3) and a second gate (R2) was defined in a sequential strategy to include CD34^+^ events. CD34^+^ cells with low SSC and low CD45 fluorescence (SSC^low^/CD45^dim^ cells) were then gated (R3) (plot 4). The low SSC properties and expression of CD45 were confirmed against total events. Finally, EPCs were identified by a gate (R4) set on a CD34 vs. CD309 (VEGFR2/KDR) dot plot and were defined as CD45^dim^/CD309^+^/CD34^+^.

To evaluate EPCs in the peripheral blood by flow cytometry, whole blood samples were labelled with monoclonal antibodies against CD34 (APC, Miltenyi Biotec), CD309 (VEGFR-2/KDR; PE, Miltenyi Biotec), and CD45 (FITC, Miltenyi Biotec), according to manufacturer’s instructions. After erythrocyte lysis, at least 250,000 CD45^+^ events were acquired on a FACS-Calibur flow cytometer (Becton Dickinson, San Jose, CA) and a minimum of 100 CD34^+^ cells were collected in each sample. Data were analysed using Paint-a-Gate software (Becton Dickinson) and the identification of the EPCs was based on morphological properties and CD45^dim^/CD309^+^/CD34^+^ profile, according to the modified ISHAGE (International Society for Hematotherapy and Graft Engineering) protocol gating strategy^43^ (Fig. 4). EPCs were reported as a percentage of leukocytes (CD45^+^ cells). The within-day coefficient of variation of EPCs quantification ranged from 3.29% to 7.47% and the intraindividual correlation was r = 0.946 (p < 0.001).

### Angiogenic factor quantification

Blood samples (7 mL) were collected by venipuncture of the antecubital vein into serum separator or EDTA-coated tubes; samples were centrifuged at 1000xg for 10 minutes or at 2000xg for 15 minutes in a refrigerated centrifuge, respectively for serum or plasma samples. Plasma and serum samples were then aliquoted and stored at −80 °C until analysis. The following biomarkers were measured, in duplicate, with enzyme-linked immunosorbent assay kits according to the manufacturer’s instructions: serum erythropoietin (IBL international GmbH, Hamburg, Germany), plasma HIF-1α (Thermo Scientific, Frederick, MD, USA), plasma SDF-1α (Thermo Scientific, Frederick, MD, USA) and plasma VEGF (IBL international GmbH, Hamburg, Germany).

### Statistical analysis

Statistical analyses were performed using IBM SPSS statistics version 21.0 (IBM Corporation, Chicago, IL, USA). Normality of the data was tested with the Shapiro-Wilk test and all data were normally distributed. Data were expressed as mean ± standard error of the mean. One-way ANOVA was used to compare group characteristics. To examine the effect of the exercise intensity on EPCs, VEGF, HIF-1α, SDF-1α and EPO, a 3 × 4 repeated-measures analysis of variance (60%/70%/80% exercise × pre-exercise/ post-exercise/6 h post-exercise/24 h post-exercise) was used to compare results between groups over time (group × time). When a significant group × time interaction was observed, post hoc means comparisons were performed using Bonferroni tests. Effect size was reported using the partial eta-squared (η^2^_p_) where values 0.01 ≤ η^2^_p_ < 0.06 represent a small effect, values 0.06 ≤ η^2^_p_ < 0.14 represent a medium effect, and values η^2^_p_ > 0.14 represent a large effect^44^. The percentage changes in EPCs and biomarkers from baseline to post-exercise, baseline to 6-hours post-exercise and baseline to 24-hours post-exercise were calculated as follows: (post-exercise value − baseline value)/baseline value × 100. The Pearson correlation was used to test associations between the changes in EPCs with the changes in VEGF, HIF-1α, SDF-1α and EPO. The level of significance was set as P ≤ 0.05.

### Data Availability

All data generated or analysed during this study are included in this published article.
